# Analysis of the Streptococcus mutans Proteome during Acid and Oxidative Stress Reveals Modules of Protein Coexpression and an Expanded Role for the TreR Transcriptional Regulator

**DOI:** 10.1128/msystems.01272-21

**Published:** 2022-03-15

**Authors:** Elizabeth L. Tinder, Roberta C. Faustoferri, Andrew A. Buckley, Robert G. Quivey, Jonathon L. Baker

**Affiliations:** a Department of Infectious Disease, St. Jude Children’s Research Hospital, Memphis, Tennessee, USA; b Center for Oral Biology, University of Rochester Medical Center, Rochester, New York, USA; c Eshelman School of Pharmacy, University of North Carolina, Chapel Hill, North Carolina, USA; d Department of Microbiology and Immunology, University of Rochester Medical Center, Rochester, New York, USA; e Genomic Medicine Group, J. Craig Venter Institute, La Jolla, California, USA; f Department of Pediatrics, UC San Diego School of Medicine, La Jolla, California, USA; NYU School of Medicine

**Keywords:** *Streptococcus mutans*, proteome, oxidative stress, Nox, TreR, trehalose

## Abstract

Streptococcus mutans promotes a tooth-damaging dysbiosis in the oral microbiota because it can form biofilms and survive acid stress better than most of its ecological competitors, which are typically health associated. Many of these commensals produce hydrogen peroxide; therefore, S. mutans must manage both oxidative stress and acid stress with coordinated and complex physiological responses. In this study, the proteome of S. mutans was examined during regulated growth in acid and oxidative stresses as well as in deletion mutants with impaired oxidative stress phenotypes, Δ*nox* and Δ*treR.* A total of 607 proteins exhibited significantly different abundances across the conditions tested, and correlation network analysis identified modules of coexpressed proteins that were responsive to the deletion of *nox* and/or *treR* as well as acid and oxidative stress. The data explained the reactive oxygen species (ROS)-sensitive and mutacin-deficient phenotypes exhibited by the Δ*treR* strain. SMU.1069-1070, a poorly understood LytTR system, had an elevated abundance in the Δ*treR* strain. S. mutans LytTR systems regulate mutacin production and competence, which may explain how TreR affects mutacin production. Furthermore, the protein cluster that produces mutanobactin, a lipopeptide important in ROS tolerance, displayed a reduced abundance in the Δ*treR* strain. The role of Nox as a keystone in the oxidative stress response was also emphasized. Crucially, this data set provides oral health researchers with a proteome atlas that will enable a more complete understanding of the S. mutans stress responses that are required for pathogenesis, and will facilitate the development of new and improved therapeutic approaches for dental caries.

**IMPORTANCE** Dental caries is the most common chronic infectious disease worldwide and disproportionately affects marginalized socioeconomic groups. Streptococcus mutans is considered a primary etiological agent of caries, with its pathogenicity being dependent on coordinated physiological stress responses that mitigate the damage caused by the oxidative and acid stress common within dental plaque. In this study, the proteome of S. mutans was examined during growth in acidic and oxidative stresses as well in *nox* and *treR* deletion mutants. A total of 607 proteins were differentially expressed across the strains/growth conditions, and modules of coexpressed proteins were identified, which enabled mapping the acid and oxidative stress responses across S. mutans metabolism. The presence of TreR was linked to mutacin production via LytTR system signaling and to oxidative stress via mutanobactin production. The data provided by this study will guide future research elucidating S. mutans pathogenesis and developing improved preventative and treatment modalities for dental caries.

## OBSERVATION

Dental caries remains the most common chronic infectious disease worldwide and is caused by a dysbiotic dental plaque microbiome that demineralizes tooth enamel via the fermentation of dietary carbohydrates to acid (1). Streptococcus mutans, a common constituent of the oral flora, is considered a primary etiological agent of caries due to its exceptional ability to facilitate biofilm formation when provided with sucrose and its acidophilic niche (2). S. mutans employs a robust acid stress response that renders it more acid tolerant than many of the health-associated commensals that it competes with ecologically. A number of these rival streptococci produce H_2_O_2_; therefore, S. mutans must also deal with oxidative stress (3, 4). Numerous studies have examined the role of various genes in these overlapping stress responses and the complex regulatory network that governs them. Previously, our research group identified that the NADH oxidase, Nox, was a linchpin of the S. mutans oxidative stress response at the intersection of two oxidative stress regulons (4). Furthermore, the transcriptional regulator of the trehalose utilization operon, TreR, had an unexpected role in oxidative stress and toxin production (5). In this study, mass spectrometry was used to elucidate changes in the S. mutans proteome during growth in acid stress and/or oxidative stress and/or upon the deletion of *nox* or *treR*.

**FIG 1:**
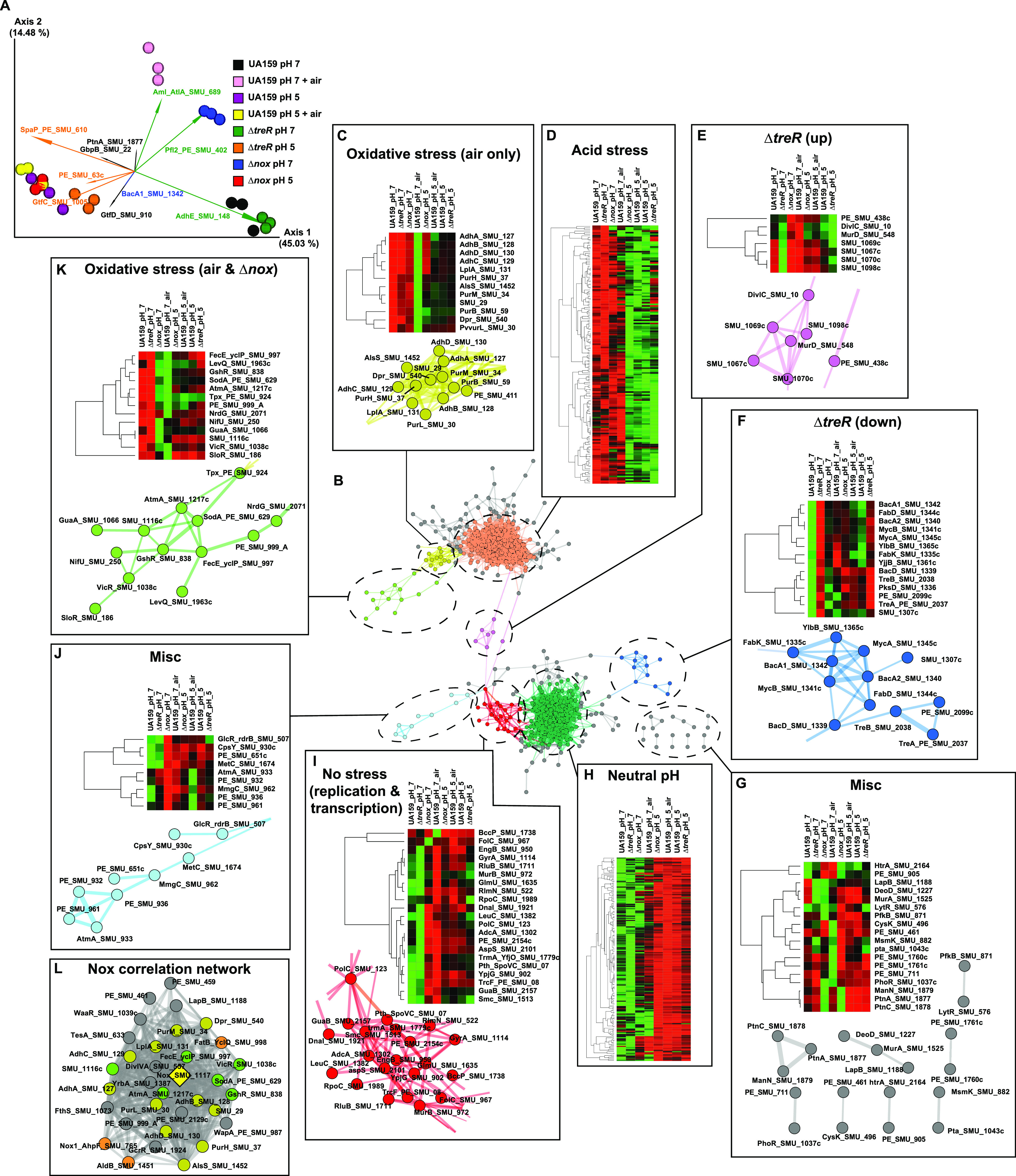
The proteome of S. mutans during acid and oxidative stress. (A) PCA biplot of the Bray-Curtis dissimilarity between samples of the indicated strains and growth conditions, represented by the colored spheres. Feature loadings (i.e., proteins that are the major drivers of the distances in ordination space) are illustrated by the vectors, which are labeled with the cognate feature name and colored based on that feature’s cluster in panel B. (B to K) A total of 607 proteins were differentially abundant across the strains and growth conditions tested, based on an uncorrected *P* value of <0.01 by analysis of variance (ANOVA). Protein coexpression was determined using Spearman’s rank correlation coefficient; only correlations with a Spearman ρ value of >0.8 are shown, and only positive correlations were considered. The central panel B shows the full correlation network of all 513 proteins that met the correlation criteria described above. Each node represents a protein, and edges (connecting lines) represent correlations of a ρ value of >0.8. Edge width is representative of Spearman’s ρ. Due to the large size and small text of the labeled full network, the labeled full network is provided in Fig. S1 in the supplemental material. The correlation network illustrates that many proteins are organized into clusters that display similar expression profiles across the strains and growth conditions, indicating that these proteins may have coregulated expression (likely in response to the mutations and/or stress conditions that were being examined). Clusters were manually selected as indicated by the node color. Panels C to K surrounding the main network show heat maps illustrating the expression profiles of the proteins in the indicated cluster of proteins across the 8 strains/growth conditions and, in most cases, also an enlarged and labeled subnetwork of the cluster. The acid-stress-associated (D) and neutral-pH-associated (H) clusters are too large to be labeled in the main text. Therefore, the labeled network for these clusters can be viewed in Fig. S1, and the labeled heat maps are provided in Fig. S2. All heat map rows are clustered by Spearman’s ρ. A pairwise correlation table of all proteins is provided in Table S2. A heat map illustrating the abundances of the 54 proteins that were differentially expressed based on ANOVA, but that did not have significant correlations with other proteins, is provided in Fig. S3. (L) Proteins that correlate with Nox when the Δ*nox* samples are not included in the network analyses. The Δ*nox* strain data likely obscured proteins that correlate with Nox; therefore, the correlation network analysis was repeated without the Δ*nox* data. The network shown here is a subcluster of all 33 proteins significantly correlating with Nox protein abundance. Nox is represented by the yellow diamond, and all other nodes are colored by the subcluster determined in panels B to K. The edge is representative of Spearman’s ρ. Only positive correlations with a ρ value of >0.8 are shown.

The archetype S. mutans strain UA159 (6), along with the Δ*nox* and Δ*treR* mutant strains, were analyzed under tightly controlled steady-state growth conditions enabled by chemostats set at neutral pH 7 or acidic pH 5 and/or sparged with air to maintain an 8.4% dissolved oxygen concentration (i.e., oxidative stress, as described in reference 4). Text S1 in the supplemental material contains a full description of the materials and methods used in this study. Liquid chromatography-tandem mass spectrometry was performed to examine the proteome of these strains and growth conditions. A total of 1,384 unique proteins were detected across the 8 strains/growth conditions (Table S1). Principal-component analysis (PCA) indicated three main clusters of samples: all pH 5 samples, regardless of oxidative stress or genotype; the pH 7 samples without oxidative stress (UA159 and Δ*treR*); and the pH 7 samples under oxidative stress (UA159 plus air and Δ*nox*) (Fig. 1A). The proteins that were the largest drivers in ordination space toward the pH 5 samples were SpaP, GtfC, GtfD, and SMU_63c, while GbpB and AdhE were associated with the pH 7 samples, and Pfl and AtlA were associated with the pH 7 samples under oxidative stress (Fig. 1A). Data from differential abundance analyses between pairwise strains/growth conditions are provided in Data Set S1. Together, these data suggest overlap in the S. mutans acid and oxidative stress responses and reveal several proteins with very large changes in abundance across the test conditions that likely are key players in the indicated stress response pathways.

10.1128/mSystems.01272-21.1 TEXT S1 Supplemental materials and methods. Download Text S1, DOCX file, 0.02 MB. Copyright © 2022 Tinder et al. 2022 Tinder et al. https://creativecommons.org/licenses/by/4.0/ This content is distributed under the terms of the Creative Commons Attribution 4.0 International license.

10.1128/mSystems.01272-21.5 TABLE S1 Normalized abundances of detected proteins. Download Table S1, XLSX file, 0.5 MB. Copyright © 2022 Tinder et al. 2022 Tinder et al. https://creativecommons.org/licenses/by/4.0/ This content is distributed under the terms of the Creative Commons Attribution 4.0 International license.

10.1128/mSystems.01272-21.8 DATA SET S1 Excel file containing pairwise log_2_ fold changes and *P* values for each protein among all 8 strains/growth conditions. Download Data Set S1, XLSX file, 5.8 MB. Copyright © 2022 Tinder et al. 2022 Tinder et al. https://creativecommons.org/licenses/by/4.0/ This content is distributed under the terms of the Creative Commons Attribution 4.0 International license.

10.1128/mSystems.01272-21.2 FIG S1 Correlation network of the S. mutans proteome. The complete network from Fig. 1B is shown, with each node labeled with the cognate protein name. Clustering of S. mutans proteins into coabundance clusters is depicted. A protein association network illustrates coexpressed proteins. Prior to clustering, proteins were filtered for significant differences using an uncorrected *P* value of <0.01 by ANOVA (607 proteins). Correlations (edges) with a Spearman ρ value of >0.8 are shown, and only positive correlations were considered. The edge width is representative of Spearman’s ρ. Clusters were manually selected as indicated by the node color. Download FIG S1, PDF file, 2.6 MB. Copyright © 2022 Tinder et al. 2022 Tinder et al. https://creativecommons.org/licenses/by/4.0/ This content is distributed under the terms of the Creative Commons Attribution 4.0 International license.

10.1128/mSystems.01272-21.3 FIG S2 Abundance profiles of proteins associated with pH 5 (A) or pH 7 (B). These are expanded versions of the heat maps appearing in Fig. 1D and H, with each row labeled. Rows are clustered by Spearman’s ρ. Download FIG S2, PDF file, 0.3 MB. Copyright © 2022 Tinder et al. 2022 Tinder et al. https://creativecommons.org/licenses/by/4.0/ This content is distributed under the terms of the Creative Commons Attribution 4.0 International license.

10.1128/mSystems.01272-21.4 FIG S3 Abundance profile of differentially expressed genes that had no significant correlations. The heat map shows the abundance of proteins that made the differential abundance cutoff of a *P* value of ≥0.5 by ANOVA but did not have any correlations with other proteins with a Spearman ρ value of ≥0.8. Rows are clustered by Spearman’s ρ. Download FIG S3, PDF file, 0.2 MB. Copyright © 2022 Tinder et al. 2022 Tinder et al. https://creativecommons.org/licenses/by/4.0/ This content is distributed under the terms of the Creative Commons Attribution 4.0 International license.

10.1128/mSystems.01272-21.6 TABLE S2 Spearman’s rank correlations between differentially expressed proteins. Download Table S2, XLSX file, 3.2 MB. Copyright © 2022 Tinder et al. 2022 Tinder et al. https://creativecommons.org/licenses/by/4.0/ This content is distributed under the terms of the Creative Commons Attribution 4.0 International license.

Correlation network analysis was performed to observe modules of coexpressed proteins under various conditions (Fig. 1B). This analysis revealed two large clusters of proteins associated with elevated abundances at either pH 5 or pH 7 (Fig. 1D and H). Several smaller subclusters were associated with other discrete protein abundance profiles, such as oxidative stress or deletion of the TreR regulator (Fig. 1C, E to G, and I to K). A cluster associated with oxidative stress, through either the addition of air or the deletion of *nox*, included many of the well-established proteins of the oxidative stress tolerance response, including Tpx, GshR, Sod, SloR, and VicR (Fig. 1K). An adjacent cluster of proteins, including the Adh operon as well as Dpr, AlsS, and much of the purine biosynthesis gene cluster, had elevated abundances at pH 7 with air but not in the Δ*nox* strain (Fig. 1C). These data further define the S. mutans oxidative stress response and clarify the proteins and pathways that are more abundant under specific conditions.

Intriguingly, two subclusters displayed abundance profiles specifically affected by the presence of the TreR regulator. DivIC and MurD, involved in cell wall synthesis and cell division, as well as the autoregulatory LytTR system SMU.1069 and SMU.1070 had increased abundances in the Δ*treR* strain (Fig. 1E). SMU.1069 and SMU.1070 exhibits cross talk with the more well-characterized LytTR systems HdrRM and BrsRM, known to regulate competence and bacteriocin production (7, 8). Since *treR* and trehalose operon expression play roles in competence (9) and the production of mutacins IV, V, and VI (5), through unknown mechanisms, signaling through SMU.1069 and SMU.1070 is an attractive hypothesis. Although the mutacin IV, V, and VI nonribosomal peptide synthetase (NRPS) products themselves are too small to be detected by the proteomics analysis employed here, further evidence linking TreR to mutacin production was observed. Several proteins within mutacin biosynthetic gene clusters (BGCs) did have significantly decreased abundances in the Δ*treR* strain, including CopYAZ (mutacin VI BGC) and SMU.1904 and SMU.1910 (mutacin V/CipB BGC) (Data Set S1). Although it remains unclear why trehalose is tied to BGC (particularly mutacin) expression in S. mutans, these data strengthen the connection and postulate that LytTR signaling is the probable link.

Meanwhile, the proteins from the trehalose operon itself as well as the large mutanobactin BGC (SMU.1334 and SMU.1349) had reduced abundances in the Δ*treR* strain (Fig. 1F). This further confirmed that in S. mutans, TreR serves as an activator of *tre* operon expression, rather than as a repressor, as seen in other species (5). Mutanobactin, a nonribosomal lipopeptide, appears to have a role in helping S. mutans deal with oxidative stress (10). Therefore, it is possible that the reduced abundance of the mutanobactin BGC may explain the impaired reactive oxygen species (ROS) tolerance in the Δ*treR* strain (5). Interestingly, Nox and TreR themselves did not appear in the correlation network, likely due to their absence in deletion mutant strains obscuring correlations. In repeated correlation analysis with the deletion mutant samples removed, the Nox abundance was tightly correlated with 33 coexpressed proteins, mainly from the clusters of genes associated with oxidative stress, further confirming its role as a keystone protein in the S. mutans oxidative stress response (Fig. 1L). Contrarily, in the reanalysis, TreR had only one protein correlation with a ρ value of ≥0.8, SMU.690. Since TreR did not exhibit a strong correlation with other proteins, but its absence had a major effect on the abundance of a number of proteins, it seems that modulation of transcriptional regulatory activity for TreR, rather than just TreR protein abundance, is likely to be key in its role as a regulator. This mechanism would allow the TreR regulon to respond directly, and very quickly, to changes in the environmental concentrations of trehalose and possibly other TreR ligands that have yet to be discovered.

**FIG 2:**
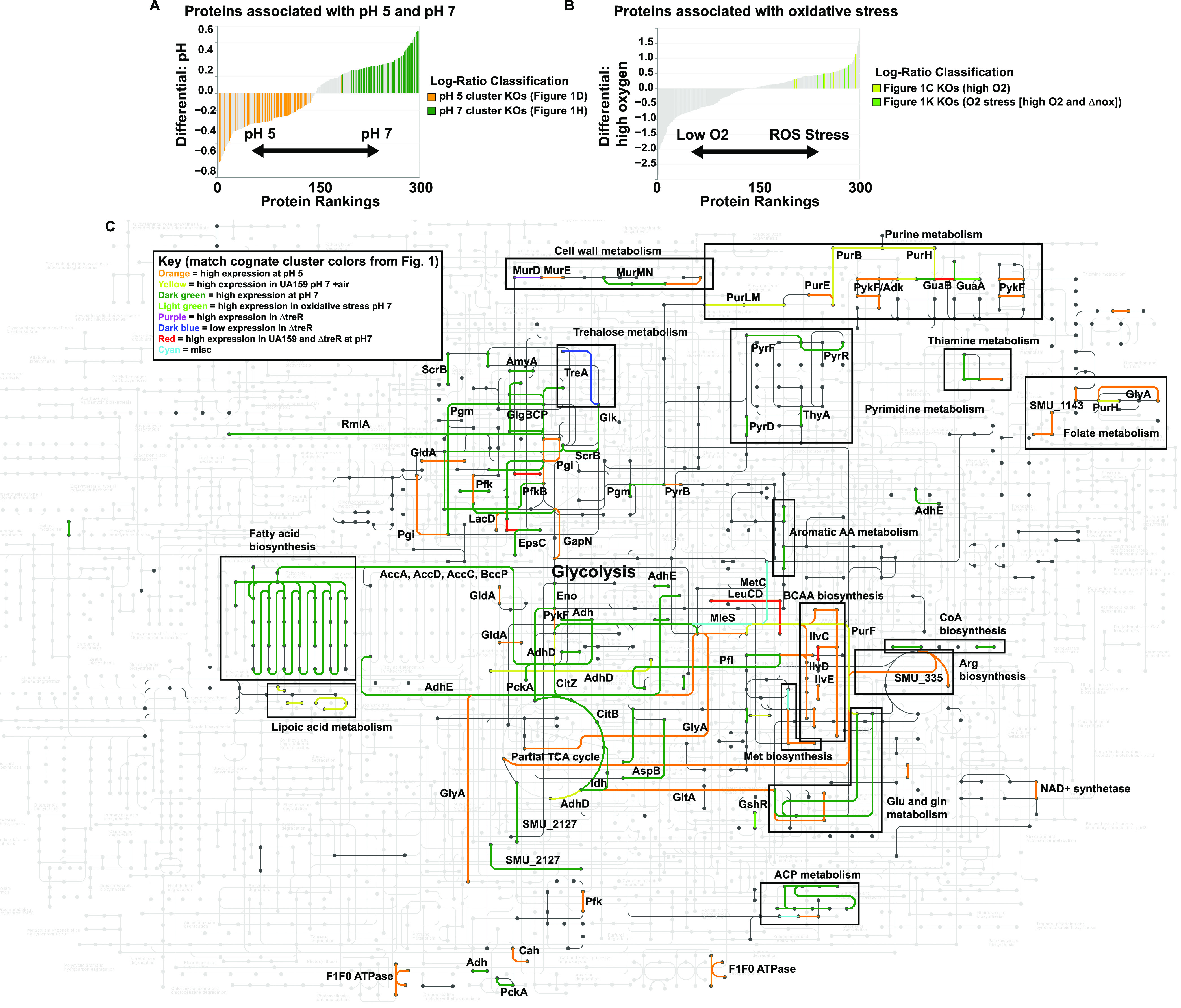
Metabolic modules of the S. mutans acid and oxidative stress responses. (A) Differential ranking of proteins associated with pH 5 versus pH 7. Songbird (11) was used to rank proteins correlated with either pH 5 or pH 7 with respect to pH, and Qurro (13) was used to visualize the resulting ranks (only the top and bottom 150 proteins are shown). Proteins with known KOs in the subclusters shown in Fig. 1D and H are highlighted in orange and dark green, respectively (the same colors used for these proteins in Fig. 1). These data indicate that the majority of the proteins that most strongly correlated with pH 5 or pH 7 are indeed found in the large clusters illustrated in Fig. 1D and H. (B) Differential ranking of proteins associated with high O_2_ (UA159 plus air and Δ*nox*) versus low O_2_ (UA159 and Δ*treR*) concentrations. Songbird was used to rank proteins with respect to high versus low O_2_ concentrations, and Qurro (13) was used to visualize the resulting ranks (only the top and bottom 150 proteins are shown). Proteins with known KOs in the subclusters shown in Fig. 1C and K are highlighted in yellow and light green, respectively (the same colors used for these proteins in Fig. 1). (C) Metabolism of S. mutans during acid and oxidative stress. All proteins from the subclusters shown in Fig. 1C to K with known KOs were overlaid onto a map of the known metabolism of S. mutans using KEGG Mapper (https://www.genome.jp/kegg). The colors of each subcluster from Fig. 1 are maintained, as described in the key. This map illustrates the components of S. mutans metabolism that are likely impacted by the differential expression of the indicated proteins across the indicated growth conditions. To reproduce an interactive version of this network, where each node and edge can be clicked on for further information, Table S3 in the supplemental material can be used as the input for the KEGG Mapper tool at https://www.genome.jp/kegg. AA, amino acid; ACP, acyl carrier protein.

Differential rankings (11) were utilized to determine the proteins most associated with acid and oxidative stress. KEGG Orthologs (KOs) from the subclusters associated with pH 5 and pH 7 (Fig. 1D and H) made up the majority of the proteins associated with the cognate pH (Fig. 2A), while proteins from the subclusters associated with oxidative stress (Fig. 1C and K) were, in fact, correlated with the associated growth conditions, based on supervised methods (Fig. 2B). To further examine the impact of the genotypes and growth conditions on S. mutans metabolism, proteins with associated KO numbers from the subclusters in Fig. 1C to J were overlaid onto a map of the metabolism of S. mutans UA159 using KEGG Mapper (https://www.genome.jp/kegg/) (Fig. 2). Table S3 provides a table of KO numbers and colors to be used by the reader to generate an interactive version of the metabolic map shown in Fig. 2C using KEGG Mapper Color. Many of the large-scale trends observed were in line with previous transcriptomic and proteomic observations (3, 12). These included increased abundances of proteins involved in fatty acid biosynthesis, the partial tricarboxylic acid (TCA) cycle, and pyrimidine metabolism at pH 7 and increased abundances of proteins involved in arginine deiminase, branched-chain amino acid (BCAA) biosynthesis, purine metabolism, and the F_1_F_o_ ATPase at pH 5. Overall, this updated perspective of the S. mutans proteome provides a comprehensive interpretation of how this organism deals with acid and oxidative stress, permitting its key role in the dysbiosis that leads to caries pathogenesis. This study also highlights several principal avenues for future research, including the importance of the TreR regulator.

10.1128/mSystems.01272-21.7 TABLE S3 KO and color list to generate an interactive S. mutans metabolism map using KEGG Mapper Color (https://www.genome.jp/kegg/mapper/color.html). Download Table S3, XLSX file, 0.01 MB. Copyright © 2022 Tinder et al. 2022 Tinder et al. https://creativecommons.org/licenses/by/4.0/ This content is distributed under the terms of the Creative Commons Attribution 4.0 International license.

### Data availability.

The raw mass spectrometry output files are available in the MassIVE Repository (https://massive.ucsd.edu) with the accession number MSV000088252.
