# Supplementary material for: Analysis of the Streptococcus mutans Proteome during Acid and Oxidative Stress Reveals Modules of Protein Coexpression and an Expanded Role for the TreR Transcriptional Regulator
Source: mSystems. 2022 Mar 15;7(2):e01272-21. doi: 10.1128/msystems.01272-21 (PMC9040809; doi:10.1128/msystems.01272-21)
Supplement: TEXT S1 [file msystems.01272-21-s0001.docx]

**Supplemental Text S1**

**Supplemental Materials and Methods.**

**Bacterial strains and growth conditions.** *Streptococcus mutans* UA159 (1), the genomic type strain, and its derivatives, ∆*treR* and ∆*nox*, were employed for this study.  Strains deleted for *treR* (2) and ∆*nox* (3) have been previously described.  Briefly, ∆*nox* was created as part of the construction of a genome-wide mutant library via a single gene deletion and replacement with an erythromycin cassette (4).  The ∆*treR* strain was created using a synthetic gene construct of the region flanking the *treR* gene, and the gene itself replaced with a kanamycin cassette (2).  The strains were grown in continuous culture in a chemostat at a dilution rate of 0.24h^-1^ in a BioFlo 2000 fermentor (New Brunswick Scientific, Edison, NJ) under glucose-limiting conditions in TY medium [3% tryptone, 0.1% (w/v) yeast extract, 0.5% (w/v) KOH, 1mM H_3_PO_4_] plus 1% (w/v) glucose, as previously described (5).  Growth at pH 7 or pH 5 was monitored by an in-dwelling pH probe (Mettler Toledo, Columbus, OH), with culture pH maintained by the addition of 2N KOH.   *S. mutans* UA159 was also grown in continuous culture to steady-state values of pH 7 and pH 5 with the addition of air to the vessel at a rate of 1 vessel volume min^-1^ (VVM), with automatic adjustment of the impeller speed to maintain a dissolved oxygen (DO) concentration of 8.4%, as measured by an in-dwelling DO probe (Mettler Toledo, Columbus, OH), as previously described (5).  Cells were removed from the fermenter at steady-state values of pH 7 and pH 5, defined as a minimum of 10 generations of continuous culture under the given condition, and collected by centrifugation.  Cell pellets were stored frozen at -80^o^C until lysed for proteome analysis.

**Preparation of whole cell lysates.**  Cell pellets were thawed on ice, and resuspended in 2mL dH_2_O.  One half of the cell suspension was utilized for the proteome analysis, while the remaining 1mL was collected by centrifugation and returned to the -80^o^C freezer.  Cells were lysed in a buffer consisting of 5% (w/v) SDS, 50mM triethylammonium bicarbonate (TEAB) (lysis buffer) containing 0.1mm glass beads in a Mini Bead Beater 8 (BioSpec Products, Bartlesville, OK).  Cell debris was removed by centrifugation at 10,000 rpm (9391 x *g*) for 10 minutes at 4^o^C.  Protein concentration was determined using a bicinchoninic acid assay kit (BCA) (Sigma Aldrich Chemical Company, St. Louis, MO).  Lysates were prepared from 3 individual steady-state cultures of each strain.

**Mass spectrometry sample preparation.**  An aliquot of 10µg of protein from each sample was removed, and brought to a volume of 25µL with lysis buffer (described above). Disulfide bonds were reduced by addition of dithiothreitol to 2mM, followed by incubation at 55°C for 1 hour. Alkylation was performed by adding iodoacetamide to 10mM and incubating at room temperature in the dark for 30 minutes.  Phosphoric acid was added to a final concentration of 1.2% from a 12% stock, followed by the addition of 6 volumes of 90% methanol, 100mM TEAB. The resulting solution was added to S-Trap micros (Protifi, Farmingdale, NY), and centrifuged at 4,000 x *g* for 1 minute. The S-Traps were washed twice with 90% methanol, 100mM TEAB.  TEAB (20 µL of 100mM stock solution) containing 1µg of trypsin was added to the S-Trap, followed by an additional 20µL TEAB. The cap to the S-Trap was loosely screwed on, but not tightened, to prevent the solution from being expelled from the S-Trap during digestion. Samples were placed in a humidity chamber at 37°C and were allowed to digest overnight, after which the S-Trap was centrifuged at 4,000 x g for 1 minute to collect the digested peptides. TFA in acetonitrile (20 µL of 0.1% stock) was added to the S-Trap, and centrifuged again.  The solutions were pooled, frozen, and dried down in a Speed Vac (Labconco, Kansas City, MO).

TMT ten-plex reagents (0.2mg) (Thermo Scientific, Rockford, IL) were removed from -20°C storage and allowed to reach room temperature prior to dissolving each tag in 20µL of acetonitrile. Samples were reconstituted in 25µL TEAB, the TMT tags were added to the samples, and incubated at room temperature for one hour. The reaction was quenched by the addition of 3µL 5% (v/v) hydroxylamine.  One-fifth (5 µg) of each sample was combined, frozen, and dried down in the Speed Vac. To increase coverage, samples were fractionated using homemade C18 spin columns. Columns were conditioned with acetonitrile, followed by equilibration with 100mM ammonium formate (AF), pH 10. The samples were resuspended in 50µL AF and added to the spin columns. After washing the columns with water and then AF buffer, samples were eluted with a gradient of acetonitrile (10%, 12.5%, 15%, 17.5%, 20%, 22.5%, 25%) followed by 50% acetonitrile in AF buffer. These fractions were then frozen, dried down, and resuspended in 0.1% TFA in water, and placed into autosampler vials.

**Mass Spectrometry Analysis.**  Peptides from each fraction were injected onto a homemade 30 cm C18 column with 1.8µm beads (Sepax, Newark, DE), with an Easy nLC-1200 HPLC (Thermo Fisher, Madison, WI), connected to a Fusion Lumos Tribrid mass spectrometer (Thermo Fisher, Madison, WI).  Solvent A was 0.1% formic acid in water, while solvent B was 0.1% formic acid in 80% acetonitrile.  Ions were introduced to the mass spectrometer using a Nanospray Flex source operating at 2 kV. The gradient began at 3% Solvent B and held for 2 minutes, increased to 10% Solvent B over 7 minutes, increased to 38% over 94 minutes, then ramped up to 90% in 5 minutes and held for 3 minutes, before returning to starting conditions in 2 minutes and re-equilibrating for 7 minutes, for a total run time of 120 minutes.  The Fusion Lumos was operated in data-dependent mode, employing the MultiNotch Synchronized Precursor Selection MS3 method to increase quantitative accuracy (6).  The cycle time was set to 3 seconds. Monoisotopic Precursor Selection (MIPS) was set to Peptide. The full scan was done over a range of 375-1500 m/z, with a resolution of 120,000 at m/z of 200, an AGC target of 4e5, and a maximum injection time of 50 ms.  Peptides with a charge state between 2-5 were picked for fragmentation. Precursor ions were fragmented by collision-induced dissociation (CID) using a collision energy of 35% and an isolation width of 0.7 m/z. MS2 scans were collected in the ion trap with an AGC target of 1e4 and a maximum injection time of 50 ms.  MS3 scans were performed by fragmenting the 10 most intense fragment ions between 400-2000 m/z, excluding ions that were 40 m/z less and 10 m/z greater than the precursor peptide, using higher energy collisional dissociation (HCD). MS3 ions were detected in the Orbitrap with a resolution of 50,000 at m/z 200 over a scan range of 100-300 m/z. The isolation width was set to 2 Da, the collision energy was 60%, the AGC was set to 1e5, and the maximum injection time was set to 105 ms. Dynamic exclusion was set to 45 seconds.

**Mass spectrometry data analysis.**  Raw data was searched using the SEQUEST search engine within the Proteome Discoverer software platform, version 2.2 (Thermo Fisher, Madison, WI), using the NCBI database.  Trypsin was selected as the enzyme allowing up to 2 missed cleavages, with an MS1 mass tolerance of 10 ppm, and an MS2 mass tolerance of 0.6 Da. Carbamidomethyl on cysteine, and TMT on lysine and peptide N-terminus were set as a fixed modification, while oxidation of methionine was set as a variable modification. Percolator was used as the FDR calculator, filtering out peptides which had a q-value greater than 0.01. Reporter ions were quantified using the Reporter Ions Quantifier node, with an integration tolerance of 20 ppm, and the integration method being set to “most confident centroid.”  Protein abundances were calculated by summing the intensities of the reporter ions from each identified peptide, while excluding any peptides with an isolation interference of 30% or more.  Normalized protein abundances are provided for each sample in Table S1.

**Filtering by ANOVA.** Proteins were filtered for significant differences across study groups as described in (7) using an uncorrected ANOVA p < 0.01 implemented in using the aov function in R v4.1.0 ([www.r-project.org](http://www.r-project.org/)).  This left 607 proteins out of the original 1,384, p-values are shown in Table S1.

**Beta diversity biplot and Qurro plots.** QIIME2 (8) was used to perform beta diversity and differential ranking analysis.  The Bray-Curtis distance metric was used to calculate beta diversity using qiime diversity beta.  PCoA analysis was performed using qiime diversity pcoa and was visualized and edited using Emperor (Figure 1A) (9).  Differential rankings for proteins associated with pH (5 vs 7) and oxygen (UA159 + air and ∆*nox* vs UA159 and ∆*treR*) were calculated using Songbird (10) and plotted using Qurro (Figure 2A and B) (11).  The QIIME visualization artifacts for the Qurro plots are provided in Files S2 and S3, and may be viewed interactively by the reader at view.qiime2.org.  Note that the Differential must be changed from ‘Intercept’ to ‘pH’ in File S2.qzv and to ‘high oxygen’ in File S3.qzv.

**Correlation network analysis.** The Spearman ρ was calculated for all protein pairs using the pwcorr function in the describedata R package.  The resulting correlation matrix was converted into a pairwise correlation table for using the melt function in the reshape2 R package (Table S2).  Only positive correlations with a ρ ≥ 0.8 were further considered.  This table was used to generate a network using Cytoscape v3.8.2 (12).  Nox and TreR did not have significant correlations in this initial analysis, likely due to the gene deletion obscuring these relationships.  These analyses were repeated, with the ∆*nox* and ∆*treR* samples removed and yielded one significant correlation for TreR, and 33 for Nox.  The network in Figure 1L was created by using Cytoscape to generate a subnetwork of all nodes, and their within-subnetwork edges, with significant correlations to Nox.  Subclusters were manually selected.  Heatmaps were generated using Morpheus (<https://software.broadinstitute.org/morpheus>). Morpheus was used to perform hierarchical clustering of rows and columns using the ‘average’ linkage method and one minus spearman ρ correlation.

**KEGG pathway analysis.** KEGG KO identifiers for the *Streptococcus mutans* UA159 proteins were obtained from the KEGG webpage ([www.genome.jp/kegg/](http://www.genome.jp/kegg/)).  Proteins from each sub-cluster identified in Figure 1 were assigned a color and plotted over the map of *S. mutans* metabolism using the KEGG Mapper – Color tool (www.genome.jp/kegg/mapper/color.html) and the ‘smu’ organism code.  Annotations were added manually using Adobe Illustrator v25.4.1.  Table S3 is a list of KO numbers and colors used to generate Figure 2C in KEGG Mapper – Color that allows the reader to generate the same interactive map.  Note that there is only one ‘green’ designation in Table S3, and that the light green, representing high expression in oxidative stress at pH 7 was manually changed from the dark green.

**Pairwise differential expression analysis.**  Simple *t*‐tests were performed using normalized abundances of proteins at pH7 vs. pH5, and also between pairs of strains assuming unequal variance among the groups. Proteins with a *p*-value cutoff < |0.05| were considered significantly abundant.  We did not employ a Log_2_ cutoff in these analyses.  This data is available in File S1.

**References**

1. Ajdic D, McShan WM, McLaughlin RE, Savic G, Chang J, Carson MB, Primeaux C, Tian R, Kenton S, Jia H, Lin S, Qian Y, Li S, Zhu H, Najar F, Lai H, White J, Roe BA, Ferretti JJ. 2002. Genome sequence of *Streptococcus mutans* UA159, a cariogenic dental pathogen. Proc Natl Acad Sci U S A 99:14434-9.

2. Baker JL, Lindsay EL, Faustoferri RC, To TT, Hendrickson EL, He X, Shi W, McLean JS, Quivey RG, Jr. 2018. Characterization of the trehalose utilization operon in *Streptococcus mutans* reveals that the TreR transcriptional regulator is involved in stress response pathways and toxin production. J Bacteriol 200.

3. Derr AM, Faustoferri RC, Betzenhauser MJ, Gonzalez K, Marquis RE, Quivey RG, Jr. 2012. Mutation of the NADH oxidase gene (*nox*) reveals an overlap of the oxygen- and acid-mediated stress responses in *Streptococcus mutans*. Appl Environ Microbiol 78:1215-27.

4. Quivey RG, Jr., Grayhack EJ, Faustoferri RC, Hubbard CJ, Baldeck JD, Wolf AS, MacGilvray ME, Rosalen PL, Scott-Anne K, Santiago B, Gopal S, Payne J, Marquis RE. 2015. Functional profiling in *Streptococcus mutans*: construction and examination of a genomic collection of gene deletion mutants. Mol Oral Microbiol 30:474-95.

5. Baker JL, Derr AM, Faustoferri RC, Quivey RG, Jr. 2015. Loss of NADH oxidase activity in *Streptococcus mutans* leads to Rex-mediated overcompensation in NAD^+^ regeneration by lactate dehydrogenase. J Bacteriol 197:3645-57.

6. McAlister GC, Nusinow DP, Jedrychowski MP, Wuhr M, Huttlin EL, Erickson BK, Rad R, Haas W, Gygi SP. 2014. MultiNotch MS3 enables accurate, sensitive, and multiplexed detection of differential expression across cancer cell line proteomes. Anal Chem 86:7150-8.

7. Wozniak JM, Mills RH, Olson J, Caldera JR, Sepich-Poore GD, Carrillo-Terrazas M, Tsai CM, Vargas F, Knight R, Dorrestein PC, Liu GY, Nizet V, Sakoulas G, Rose W, Gonzalez DJ. 2020. Mortality risk profiling of *Staphylococcus aureus* bacteremia by Multi-omic serum analysis reveals early predictive and pathogenic signatures. Cell 182:1311-1327 e14.

8. Bolyen E, Rideout JR, Dillon MR, Bokulich NA, Abnet CC, Al-Ghalith GA, Alexander H, Alm EJ, Arumugam M, Asnicar F, Bai Y, Bisanz JE, Bittinger K, Brejnrod A, Brislawn CJ, Brown CT, Callahan BJ, Caraballo-Rodriguez AM, Chase J, Cope EK, Da Silva R, Diener C, Dorrestein PC, Douglas GM, Durall DM, Duvallet C, Edwardson CF, Ernst M, Estaki M, Fouquier J, Gauglitz JM, Gibbons SM, Gibson DL, Gonzalez A, Gorlick K, Guo J, Hillmann B, Holmes S, Holste H, Huttenhower C, Huttley GA, Janssen S, Jarmusch AK, Jiang L, Kaehler BD, Kang KB, Keefe CR, Keim P, Kelley ST, Knights D, et al. 2019. Reproducible, interactive, scalable and extensible microbiome data science using QIIME 2. Nat Biotechnol 37:852-857.

9. Vazquez-Baeza Y, Pirrung M, Gonzalez A, Knight R. 2013. EMPeror: a tool for visualizing high-throughput microbial community data. Gigascience 2:16.

10. Morton JT, Marotz C, Washburne A, Silverman J, Zaramela LS, Edlund A, Zengler K, Knight R. 2019. Establishing microbial composition measurement standards with reference frames. Nat Commun 10:2719.

11. Fedarko MW, Martino C, Morton JT, Gonzalez A, Rahman G, Marotz CA, Minich JJ, Allen EE, Knight R. 2020. Visualizing 'omic feature rankings and log-ratios using Qurro. NAR Genom Bioinform 2:lqaa023.

12. Shannon P, Markiel A, Ozier O, Baliga NS, Wang JT, Ramage D, Amin N, Schwikowski B, Ideker T. 2003. Cytoscape: a software environment for integrated models of biomolecular interaction networks. Genome Res 13:2498-504.
