# Supplementary figures and images for: Analysis of the Streptococcus mutans Proteome during Acid and Oxidative Stress Reveals Modules of Protein Coexpression and an Expanded Role for the TreR Transcriptional Regulator
Source: mSystems. 2022 Mar 15;7(2):e01272-21. doi: 10.1128/msystems.01272-21 (PMC9040809; doi:10.1128/msystems.01272-21)

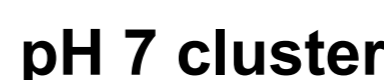

# B

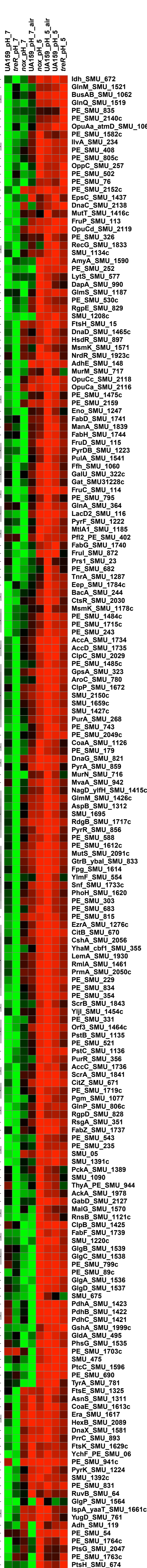

Supplement: FIG S2 [file msystems.01272-21-sf002.pdf]

Figure S3

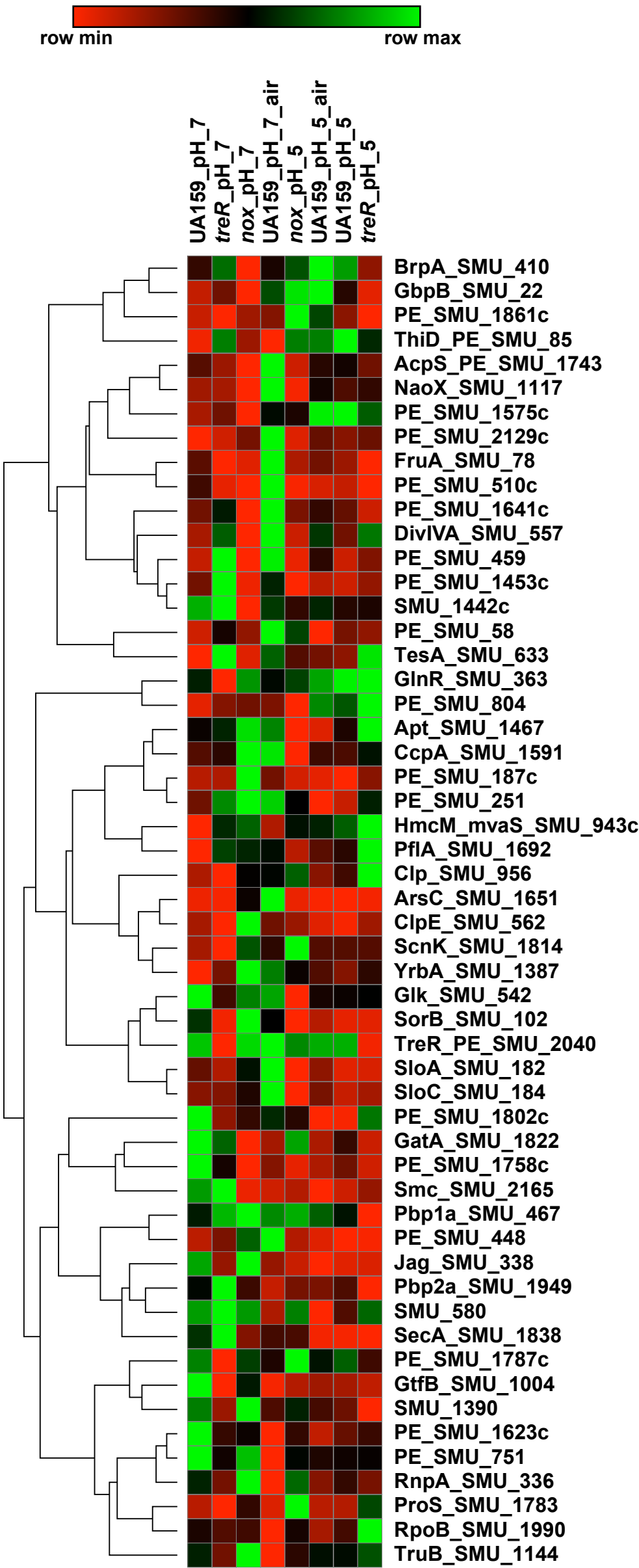

Supplement: FIG S3 [file msystems.01272-21-sf003.pdf]
