# Supplementary material for: Analysis of the Streptococcus mutans Proteome during Acid and Oxidative Stress Reveals Modules of Protein Coexpression and an Expanded Role for the TreR Transcriptional Regulator
Source: mSystems. 2022 Mar 15;7(2):e01272-21. doi: 10.1128/msystems.01272-21 (PMC9040809; doi:10.1128/msystems.01272-21)
Supplement: FIG S1 [file msystems.01272-21-sf001.pdf]

Figure S1

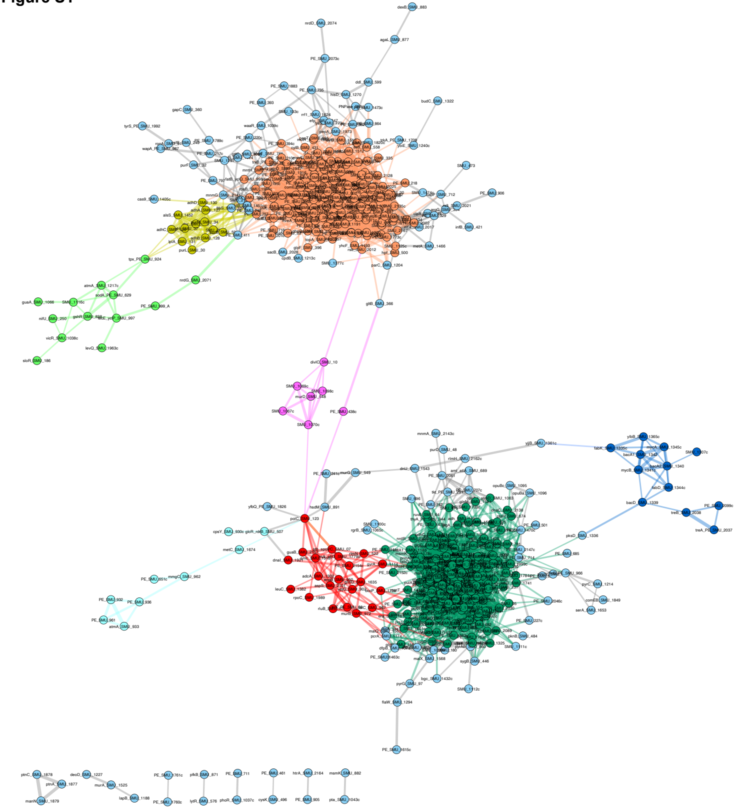
dexB_SMU_883
nrdD_SMU_2074
agaL_SMU_877
PE_SMU_2073c
ddl_SMU_599
PE_SMU_1883
PE_SMU_735
hisD_SMU_1270
budC_SMU_1322
PE_SMU_393
gapC_SMU_360
tyrS_PE_SMU_1992
waaR_SMU_1039c
PE_SMU_220c
PE_SMU_217c
purF_SMU_32
PE_SMU_797
SMU_473
PE_SMU_906
cas9_SMU_1405c
adhD_SMU_130
alsS_SMU_1452
adhC_SMU
lplA_SMU_131
purL_SMU_30
adhB_SMU_128
infB_SMU_421
metA_SMU_1466
hpf_SMU_500
sacB_SMU_2028
cpdB_SMU_1213c
SMU_1377c
parC_SMU_1204
tpx_PE_SMU_924
nrdG_SMU_2071
atmA_SMU_1217c
sodA_PE_SMU_629
guaA_SMU_1066
SMU_1116c
PE_SMU_899_A
nifU_SMU_250
gshR_SMU_838
levQ_SMU_1963c
vicR_SMU_1038c
sloR_SMU_186
gltB_SMU_366
divIC_SMU_10
SMU_1069c
SMU_1098c
murD_SMU_548
SMU_1067c
PE_SMU_438c
SMU_1070c
mnmA_SMU_2143c
purD_SMU_48
yjjB_SMU_1361c
ylbB_SMU_1365c
fabK_SMU_1335c
mycA_SMU_1345c
bacA1_SMU_1342
SMU_1307c
bacA2_SMU_1340
mycB_SMU_1341c
fabD_SMU_1344c
bacD_SMU_1339
PE_SMU_2099c
treB_SMU_2038
treA_PE_SMU_2037
rlmH_SMU_2162c
dnlJ_SMU_1543
aml_atlA_SMU_689
PE_SMU_241c
murG_SMU_549
PE_SMU_2061
opuBc_SMU_1095
opuBa_SMU_1096
hsdM_SMU_891
ylfQ_PE_SMU_1826
polC_SMU_123
cpsY_SMU_930c
glcR_rdrB_SMU_507
metC_SMU_1674
pksD_SMU_1336
PE_SMU_685
PE_SMU_966
pyrC_SMU_1214
comEB_SMU_1849
serA_SMU_1653
PE_SMU_651c
mmgC_SMU_962
PE_SMU_932
PE_SMU_936
PE_SMU_961
atmA_SMU_933
dnaI_SMU_1923
leuC_SMU_1382
rpoC_SMU_1989
rluB_SMU
murB_SMU_972
PE_SMU_2046c
PE_SMU_227c
pknB_SMU_484
SMU_1111c
sygB_SMU_446
malX_SMU_1568
bgc_SMU_1432c
SMU_1112c
pyrG_SMU_97
flaW_SMU_1294
PE_SMU_1615c
PE_SMU_1463c
pcrA_SMU_1174
ptnC_SMU_1878
ptnA_SMU_1877
manN_SMU_1879
deoD_SMU_1227
murA_SMU_1525
lapB_SMU_1188
PE_SMU_1761c
PE_SMU_1760c
pfkB_SMU_871
lytR_SMU_576
PE_SMU_711
phoR_SMU_1037c
PE_SMU_461
cysK_SMU_496
htrA_SMU_2164
PE_SMU_905
msmK_SMU_882
pta_SMU_1043c
